# Activation of bacterial transcription by distortion of promoter base pairing

**DOI:** 10.1093/nar/gkaf1424

**Published:** 2026-01-08

**Authors:** Ksenia M Klimova, David Forrest, Prateek Sharma, James R J Haycocks, David C Grainger

**Affiliations:** School of Biosciences, University of Birmingham, Edgbaston, Birmingham B15 2TT,United Kingdom; School of Biosciences, University of Birmingham, Edgbaston, Birmingham B15 2TT,United Kingdom; School of Biosciences, University of Birmingham, Edgbaston, Birmingham B15 2TT,United Kingdom; School of Biosciences, University of Birmingham, Edgbaston, Birmingham B15 2TT,United Kingdom; School of Biosciences, University of Birmingham, Edgbaston, Birmingham B15 2TT,United Kingdom

## Abstract

Bacterial RNA polymerase binds and unwinds promoter DNA to initiate transcription. The process is often inefficient and can be stimulated by activator proteins. For example, many activators bind RNA polymerase and promoters simultaneously, stabilizing their interaction. Working with the multiple antibiotic resistance activator (MarA) protein of *Escherichia coli*, we have discovered an alternative mechanism. We show that, when bound upstream of the *flgBCDEFGHIJ/KL* operon, MarA perturbs base pairing adjacent to its DNA target. This compensates for inefficient duplex unwinding by RNA polymerase and, as a result, activates transcription. Consistent with our model, an appropriate base pair mismatch mimics the effect of, and removes the need for, MarA. As many regulators alter DNA conformation, we suggest that this mechanism of activation could be commonplace.

## Graphical Abstract

**Graphical Abstract:**
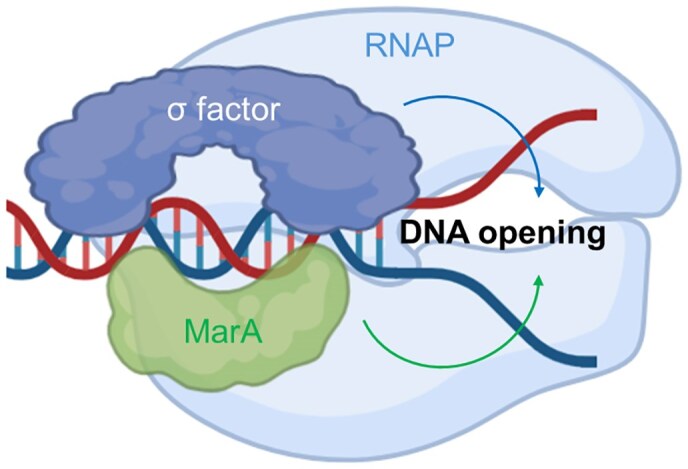

## Introduction

In bacteria, sequence specific transcription initiation requires a dissociable RNA polymerase subunit called the σ factor [1]. In *Escherichia coli*, the housekeeping σ^70^ factor interacts with two promoter DNA elements, named according to their position with respect to the transcription start site (TSS, +1) [2]. These hexameric DNA sequences have different roles. The −35 element is needed for initial RNA polymerase binding [3], while the −10 sequence, positioned 17 bp downstream, is unwound to allow DNA template strand recognition [4, 5]. Unwinding initiates when a near universal non-template strand adenine, at position −11, unpairs and flips out of the DNA base stack [5]. This step is enhanced by consecutive tryptophan side chains in σ^70^, which act as a wedge to separate the DNA strands [5]. Subsequently, the W-dyad is reconfigured, and a σ-factor pocket captures the unpaired A_−11_. This defines the upstream boundary of DNA melting and promotes further unwinding downstream [4]. Thus, strand separation proceeds towards the TSS until a complete transcription bubble, around 13 nucleotides in length, is formed [4]. The *E. coli* genome encodes six alternative σ factors that recognize distinct promoter sequences [6–10]. Most σ variants are related to σ^70^ but may lack one, or in some cases both, W-dyad residues that stimulate DNA melting [11]. To compensate, alternative σ factors often require promoters that closely match the consensus sequence [11].

Many promoters are inefficient in the absence of activator proteins [1]. Often, these regulators simultaneously contact the transcriptional apparatus, and promoter DNA, to stabilize their interaction [1, 12]. The order in which such complexes assemble can differ. For instance, some factors bind the promoter and then ‘recruit’ RNA polymerase [12]. Others ‘pre-recruit’ the enzyme, as an activator–RNA polymerase complex, that scans DNA for a suitable promoter [13–15]. In both scenarios, the regulator binds DNA upstream of the core promoter elements [16]. Occasionally, activators bind within the core promoter and the regulatory mechanism is different [17]. For example, MerR family proteins bind between promoter −10 and −35 hexamers that are too far apart [18]. As a result, the DNA is kinked and promoter element spacing is optimized [18]. Regardless of mechanism, almost all activators link environmental cues to changes in gene expression [1].

The multiple antibiotic resistance activator (MarA) protein is made in response to extracellular stress [19–21]. Consequently, by means of pre-recruitment, σ^70^-dependent genes are upregulated [13, 21]. In some cells, mutations cause constitutive MarA production. The subsequent changes in gene expression drive antibiotic resistance [20]. Thus, the identity of MarA-controlled genes is of great interest. However, MarA binds a degenerate DNA target that can be shared by related activators [14, 22–24]. Hence, detection of such regulatory interactions is difficult. In recent work, we combined experimental and bioinformatic tools to better predict MarA targets in *E. coli* [25]. This identified putative MarA sites upstream of genes for flagellar production [25]. In this work, we have used biochemistry and genetics to test our predictions. Unexpectedly, we show that a promoter requiring the alternative σ^28^ factor is MarA activated. The mechanism of activation is unusual; MarA binds within, rather than upstream of, the core promoter. Consequently, base pairing is disrupted at precise positions on either side of the MarA target. At the promoter in question, this impacts the non-template strand base A_−11_, which is inefficiently unpaired by the σ^28^ RNA polymerase alone. Consistent with our model, if A_−11_ cannot base pair, because of a template strand mutation, the requirement for MarA is removed. Thus, MarA stimulates duplex unwinding to activate transcription.

## Materials and methods

### Strains, plasmids, and oligonucleotides

Strains, plasmids, and oligonucleotides are listed in Supplementary Table S1. Standard procedures were used throughout.

### ß-galactosidase assays

Strains encoding promoter::*lacZ* fusions in plasmid pRW50 were grown aerobically at 37°C in Lennox broth (LB) medium until reaching mid-log phase. Cells were lysed with toluene and 1% (*w/v*) sodium deoxycholate. To measure β-galactosidase activity, we followed the method described by Miller [26]. For each assay, at least three biological replicates were used and data shown are the mean of these experiments. Error bars show standard deviation.

### Protein purification

MarA was overproduced from plasmid pET28a-*marA* in T7 express cells, following the protocol of Kettles *et al.* [27]. After overexpression, cells were collected by centrifugation and resuspended in 50 mM Tris–HCl (pH 7.5), 1 mM ethylenediaminetetraacetic acid (EDTA), and 1 M NaCl. After lysis by sonication, inclusion bodies were collected by centrifugation and resuspended in 50 mM Tris–HCl (pH 8.5), 6 M guanidinium-HCl, and loaded onto a pre-charged HisTrap Ni Sepharose High Performance column. Unbound protein was washed from the column with Buffer A [1M NaCl, 50 mM Tris–HCl (pH 8.5)]. Bound protein was then eluted with a linear gradient of Buffer B (Buffer A + 1 M imidazole). The MarA-containing fractions were dialyzed against Buffer X [1 M NaCl, 50 mM 4-(2-Hydroxyethyl)-1-piperazineethanesulfonic acid (HEPES), 1mM Dithiothreitol (DTT), 5 mM EDTA, 0.1 mM Triton X-100, 20% (*v/v*) glycerol] for storage at −80°C.

FlhDC was overproduced from pET26b-*flhDC* in T7 express cells. After overexpression, cells were collected by centrifugation and resuspended in Buffer A + 1 tablet of EDTA-free protease inhibitor cocktail + 0.1 mM DTT. After lysis by sonication, and clearing by centrifugation, the supernatant was loaded onto a HiTrap Heparin column. The column was washed with Buffer A and then eluted with a NaCl gradient up to 1 M. The FlhDC containing fractions were then dialyzed against Buffer A and loaded onto a pre-equilibrated HiTrap Q anion-exchange column. After elution using a NaCl gradient, fractions containing FlhDC were concentrated using Vivaspin columns and loaded onto a pre-equilibrated size-exclusion column (Sephacryl S-200) in Buffer C [0.1 M NaCl, 20 mM Tris–HCl (pH 7.9)]. Fractions containing FlhDC were combined and transferred into storage buffer [0.1 M NaCl, 20 mM Tris–HCl (pH 7.9), 50% (*v/v*) glycerol, 0.1 mM DTT, 0.1 mM EDTA] by dialysis for storage at −80°C.

To purify σ^28^, we used the method of Hollands *et al.* [28]. RNA polymerase core enzyme and RNA polymerase σ^70^ holoenzyme were purchased from NEB. RNA polymerase σ^28^ holoenzyme was generated by incubating excess σ factor with the core enzyme at 37°C for 10 min before use.

### Making double stranded DNA fragments with a base pairing mismatch

Oligonucleotide pairs (1 μmol each) were mixed in annealing buffer (30 mM HEPES, pH 7.5, and 100 mM potassium acetate) and incubated at 92°C for 5 min. Subsequently, the mixture was cooled slowly to 4°C and unannealed oligonucleotides were removed by agarose gel electrophoresis.

### DNAse I footprinting

DNA fragments were excised from pSR using *Aat*II and *Hin*dIII. After end-labelling with γ32-ATP and T4 PNK (NEB), footprints were done as previously described in buffer containing 40 mM Tris acetate (pH 7.9), 50 mM KCl, 5 mM MgCl_2_, 500 μM DTT, and 12.5 μg/ml Herring Sperm DNA [29, 30]. Resulting DNA fragments were analysed on a 6% denaturing gel. Subsequently, dried gels were exposed to a Biorad phosphor screen that was scanned using a Bio-Rad Molecular Imager FX.

### KMnO_4_ footprinting

For KMnO_4_ footprinting assays, a [γ^32^P]-ATP radiolabelled DNA fragment was used. All reactions were done at 37°C in 40 mM Tris acetate (pH 7.9), 1 mM MgCl_2_, 0.1 M KCl, 1 mM DTT, and 100 µg/ml bovine serum albumin (BSA). The final reaction volume was 20 µl. After 30 min, 1 µl of 200 mM KMnO_4_ solution was added to the sample and incubated at 37°C for 4 min. The reactions were stopped by adding 50 µl of stop solution (3.0 M NH_4_CH_3_CO_2_, 0.1 mM EDTA, 1.5 M 2-mercaptoethanol). To each reaction, 130 µl of Tris-EDTA (TE) buffer was added and DNA was extracted with 200 µl of phenol–chloroform. The DNA was then precipitated with 70% (*v/v*) ethanol and GlycoBlue at −70 °C, before being collected by centrifugation at 6°C. After the removal of ethanol, pellets were dried, resuspended in 1 M piperedine, and incubated at 90°C for 30 min. The reactions were stopped with 10 µl of 3M sodium acetate, 300 µl of 100% (*v/v*) ethanol, and 1 µl GlycoBlue, before precipitation at −70°C. The DNA was collected by centrifugation and washed twice with 70% (*v/v*) ethanol. The supernatant was then removed and the pellet dried before being resuspended in 10 µl of DNAseI blue loading dye [5 M urea, 20 mM NaOH, 1 mM EDTA, 0.025% (*v/v*) bromophenol blue, 0.025% (*v/v*) xylene cyanol FF].

Prior to loading, all samples were heated to 80°C for 2 min. Labelled DNA fragments were analysed on a 6% denaturing polyacrylamide gel and visualized using a Fuji phosphor screen and Bio-Rad Molecular Imager FX.

### Electrophoretic mobility shift assays

Experiments were done as described by Grainger *et al.* [31]. Briefly, radiolabelled DNA fragments were used at a final concentration of ∼10 nM. Note that all *in vitro* DNA binding reactions contained a vast excess (12.5 µg/ml) of Herring sperm DNA as a non-specific competitor. Footprints were analysed on a 6% DNA sequencing gel (Molecular Dynamics). Prior to loading, all DNAse I footprinting samples were heated to 80°C for 2 min. Labelled DNA fragments were analysed on a 6% denaturing polyacrylamide gel and visualized using a Fuji phosphor screen and Bio-Rad Molecular Imager FX.

### *In vitro* transcription assays

The *in vitro* transcription experiments were done as described previously [32]. A Qiagen maxiprep kit was used to purify pSR plasmid carrying the different promoter inserts. This template (∼16 µg/ml) was pre-incubated with purified MarA or/and FlhDC in buffer containing 0.2 mM cyclic AMP (cAMP), 20 mM Tris (pH 7.9), 5 mM MgCl_2_, 500 µM DTT, 50 mM KCl, 100 µg/ml BSA, 200 µM ATP, 200 µM GTP, 200 µM CTP, and 10 µM UTP with 5 µCi [α-^32^P]-UTP. The reaction was initiated by adding the appropriate RNA polymerase holoenzyme and incubated at 37°C for 20 min. Labelled RNA products were analysed on a 6% (*w/v*) denaturing polyacrylamide gel and visualized using a Fuji phosphor screen and Bio-Rad Molecular Imager FX. Band intensities were determined using Quantity One software.

### Structural modelling

Structural modelling was done with ChimeraX using the σ^28^-holoenzyme–promoter open complex structure [33] and the MarA–DNA structure [34] (PDB accession numbers 6PMI and 1BL0, respectively). In Chimera X, the *flgB*P2 sequence was mapped onto the DNA sequence of 6PMI by aligning the promoter motifs recognized by σ^28^. In doing so, we also identified which 6PMI bases correspond to the *flgB*P2 marbox (i.e. positions −16 to −30, relative to the TSS). Hence, the marbox of 1BL0 was aligned with 6PMI on this basis. Next, since MarA bends the DNA by 35°, we manually distorted DNA in the 6PMI structure to match the DNA trajectory in 1BL0. Last, we removed DNA present in 1BL0 to leave MarA correctly docked on the 6PMI structure.

## Results

### The *Escherichia coli* MarA protein binds DNA targets adjacent to genes needed for flagellar biosynthesis

**Figure 1.**
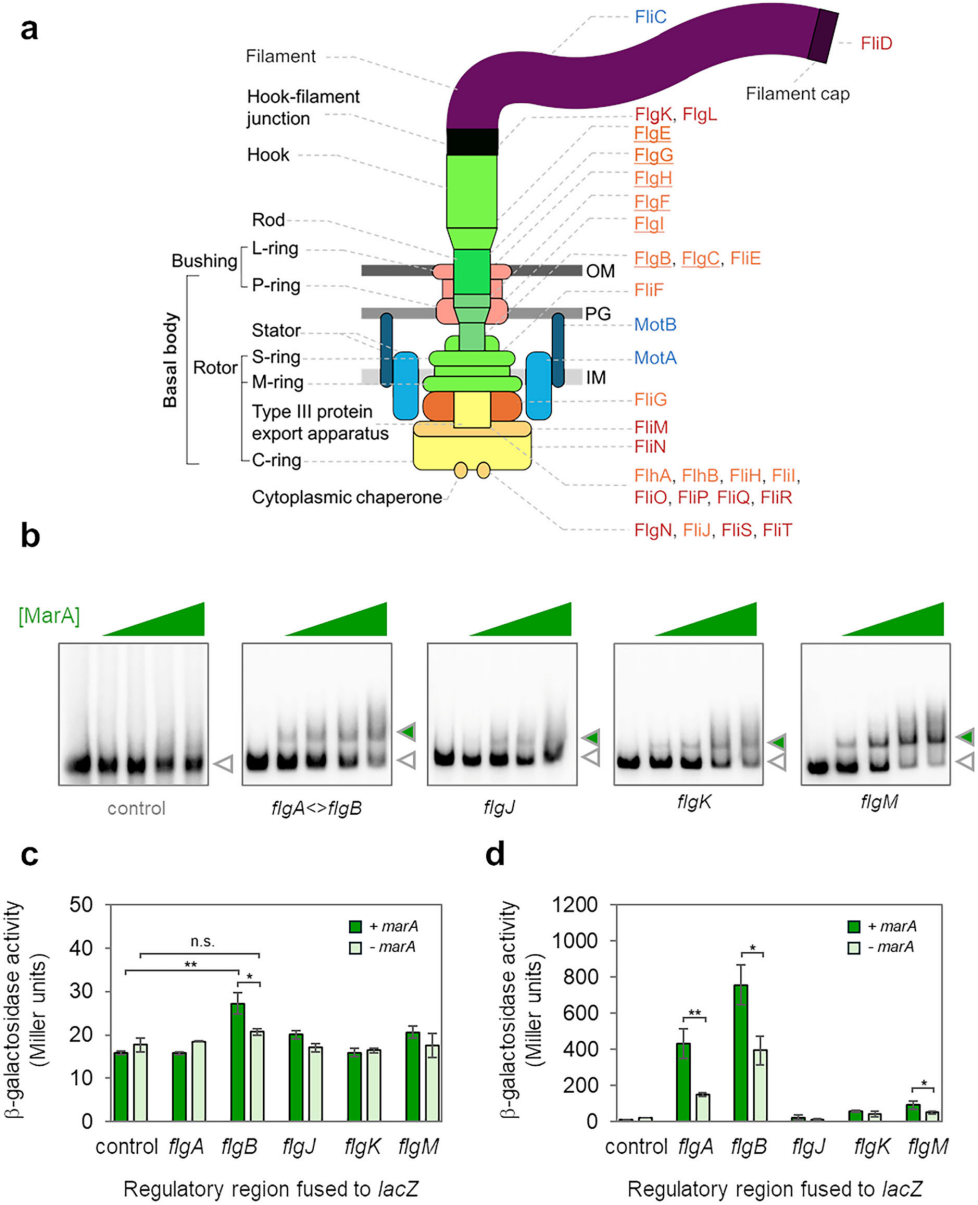
The MarA protein targets regulatory regions of genes encoding flagellar subunits. (**a**) Schematic representation of the *Escherichia coli* flagellar complex. Structural components of the complex, and substructures thereof, are labelled in black. Individual proteins are labelled according to the role of FlhD_4_C_2_ (orange), σ^28^ (blue), or both (red) in controlling their expression [35]. Underlining indicates a role for σ^28^ is suggested by this work, in addition to a known dependence on FlhD_4_C_2_. (**b**) Binding of purified MarA to regulatory regions upstream of genes encoding flagellar subunits. Each image shows the result of an EMSA with purified MarA protein (0.25, 0.5, 0.75, or 1 μM). The control DNA fragment, not expected to bind MarA, is a section of the *hapR* promoter region from *Vibrio cholerae* [30]. Bands corresponding to free and MarA-bound DNA fragments are labelled with open and filled triangles, respectively. (**c**) Promoter activity in the absence of ectopic FlhD_4_C_2_ expression. The indicated regulatory DNA fragments were cloned upstream of *lacZ* in plasmid pRW50 and the resulting constructs used to transform *E. coli* JCB387 cells. Where indicated, MarA was expressed ectopically from plasmid pJ203. Values are the mean of three independent experiments and error bars indicate standard deviation. A two-tailed and paired Student’s *t*-test was used to calculate *P* (* <.05 and ** <.01). (**d**) Promoter activity in the presence of ectopic FlhD_2_C_2_ expression. As in panel (c) except that cells also expressed FlhD_4_C_2_ from plasmid pUC19. Values are the mean of at least three independent experiments and error bars indicate standard deviation. A two-tailed and paired Student’s *t*-test was used to calculate *P* where appropriate; otherwise, a two-tailed homoscedastic test was used (* <.05, ** <.01 and *** <.001).

In *E. coli*, and related bacteria, flagellar production involves a well-defined gene regulatory cascade (controlled genes are shown in Fig. 1a) [35, 36]. The trigger is expression of the *flhDC* operon, encoding a heterohexameric activator called FlhD_4_C_2_ [37]. This stimulates σ^70^-bound RNA polymerase to transcribe genes encoding the flagellar hook, basal body, and ancillary factors [35, 38, 39]. Additionally, the σ^28^ factor, an alternative RNA polymerase subunit related to σ^70^, is expressed [40]. Subsequently, promoters recognized by σ^28^ drive expression of remaining flagellar components [41, 42]. These form a capped filament and a junction linking this to the hook [43]. There is substantial crosstalk between phases of the cascade [35]. Hence, regulatory regions bound by FlhD_4_C_2_ and σ^70^ may also contain promoters used by σ^28^. Additionally, in our recent work, we identified putative targets for MarA within the regulatory pathway for flagellar production [25]. Here, we wanted to understand whether MarA could bind the predicted targets specifically. To test this, we generated a set of DNA fragment encompassing each potential site and measured MarA binding using electrophoretic mobility shift assays (EMSAs). As a control, we used a nucleic acid sequence that does not bind MarA, the enterotoxigenic *E. coli estA* promoter [44]. The results are shown in Fig. 1b. As expected, MarA did not bind to the control sequence. Conversely, MarA altered the migration of all DNA fragments containing a predicted target site.

### Production of MarA stimulates *flgAMN* and *flgBCDEFGHIJ/KL* expression *in vivo*

To understand whether MarA could impact transcription, we fused each regulatory DNA fragment to *lacZ* in plasmid pRW50. The resulting DNA constructs were used to transform *E. coli* cells, containing plasmid pJ203, or a derivative that expresses MarA, avoiding the need to induce *marRAB* with toxic compounds. The β-galactosidase assay data are shown in Fig. 1c. Most regulatory DNA fragments were inactive, likely because FlhD_4_C_2_ is scarce in the conditions of our experiment. Only transcription from the *flgB* regulatory DNA was significantly above background levels and increased in the presence of MarA (Fig. 1c). Next, we repeated the experiment in cells also harbouring a pUC19 variant encoding FlhD_4_C_2_ (Fig. 1d). Consistent with our interpretation, with FlhD_4_C_2_ present, all DNA fragments, except the region upstream of *flgJ*, stimulated transcription significantly. We conclude that there is no promoter upstream of *flgJ*. The most notable impact of MarA was on transcription from the *flgA* and *flgB* regulatory DNA. We also observed increased *flgM* expression, in the presence of ectopic *marA*, but have not studied this further.

### MarA binding to the intergenic region between the divergent *flgAMN* and *flgBCDEFGHIJ/KL* operons distorts DNA base pairing

**Figure 2.**
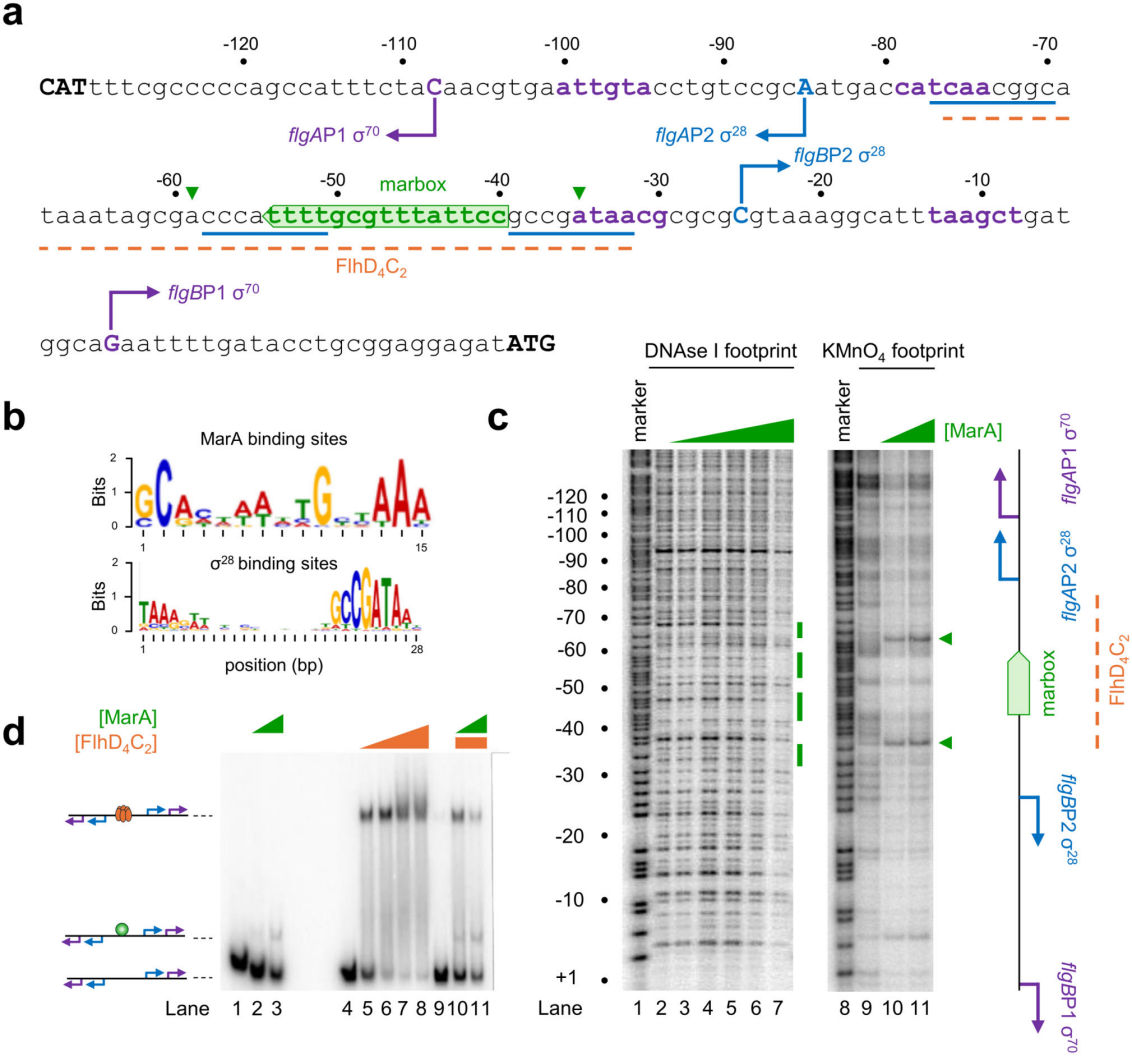
Organization of regulatory DNA between the divergent genes *flgA* and *flgB*. (**a**) Sequence of the regulatory DNA between the divergent genes *flgA* and *flgB*. All numbering is with respect to the *flgA*P1 TSS (+1). Sites of transcription initiation are in upper case and marked by bent arrows. Promoter −10 and −35 elements for σ^70^ binding are in purple and the equivalent regions for σ^28^ are underlined blue. Note that *flgA*P2 and *flgB*P2 share the same −35 element. The binding site for FlhD_4_C_2_ is underlined orange and the position and orientation of the MarA target is shown by the green block arrow. (**b**) DNA sequence motifs for MarA and σ^28^ binding derived from ChIP-seq experiments. DNA sequence logos generated from sets of targets for MarA and σ^28^ identified using ChIP-seq [21, 35]. (c) MarA binding disrupts pairing of bases on either side of its target site. The images show results of DNAse I (lanes 2–7) or KMnO_4_ (lanes 9–11) footprinting assays. The gels are calibrated with Maxam–Gilbert GA sequencing reactions (lanes 1 and 8) and all numbering is with respect to the *flgB*P1 TSS (+1). Where present, MarA was added at concentrations of 0.5, 1, 2, 2.5, 3 μM (lanes 3–7) and 0.5 or 3 μM (lanes 10 and 11). Regions of protection from DNAse I digestion, and sensitivity to attached by KMnO_4_, are shown by green bars or triangles, respectively. (**d**) MarA and FlhD_4_C_2_ compete to bind overlapping target sites. Results of an EMSA with different concentrations of FlhD_4_C_2_ (0.05, 0.1, 0.2, 0.25 µM) and MarA (0.5, 1, 1.5, 2 µM). In lanes 10 and 11, 0.05 µM of FlhD_4_C_2_ was used, as well as 0.05 and 0.1 µM of MarA, respectively. The cartoon schematic illustrates different configurations of bound proteins.

The *flgAMN* and *flgBCDEFGHIJ/KL* operons are divergent and so have overlapping regulatory regions. Previous work identified oppositely orientated σ^70^-dependent promoters (*flgA*P1 and *flgB*P1) at the locus [45]. Both promoters are activated by FlhD_4_C_2_, which binds a single site. The different sequence elements are shown in Fig. 2a, alongside the predicted binding site for MarA. Note that the MarA target is in the reverse orientation and completely overlaps the site for FlhD_4_C_2_. For comparison, the DNA consensus for MarA binding is shown in Fig. 2b (top panel). To understand whether MarA recognizes the predicted site, we first used DNAse I footprinting. The results are shown in Fig. 2c (lanes 1–7). As is typical for MarA, the footprint is subtle [Sharma *et al.* [21] and Robert G. Martin (personal communication)]. Even so, the DNA protection observed locates exactly to the expected region. We reasoned that other footprinting approaches might be more sensitive to MarA binding. Hence, we used KMnO_4_ footprinting, where unpaired thymine bases ultimately cause DNA strand cleavage [46]. The result of the experiment is shown in Fig. 2c (lanes 8–11). Addition of MarA causes a marked increase in DNA cleavage, at equivalent positions, 5 base pairs on either side of the MarA binding site. These locations are marked by green triangles in Fig. 2a and c. We conclude that MarA binds the predicted site and impacts DNA base pairing at adjacent locations, most likely by bending the double helix [34].

### MarA and FlhDC compete for binding to an overlapping target site

We reasoned that MarA and FlhD_4_C_2_ might compete to recognize their overlapping target sites. To test this, we again used EMSAs (Fig. 2d). Addition of MarA retarded DNA migration as expected (lanes 1–3). Similarly, FlhD_4_C_2_ altered DNA mobility but to a much greater extent (lanes 4–8). In the presence of MarA, abundance of the FlhD_4_C_2_–DNA complex decreased and a band indicative of MarA binding alone was detected (lanes 9–11). We conclude that MarA and FlhD_4_C_2_ cannot recognize their overlapping DNA targets simultaneously.

### MarA does not activate σ^70^-dependent transcription from *flgA*P1 or *flgB*P1 *in vitro*

**Figure 3.**
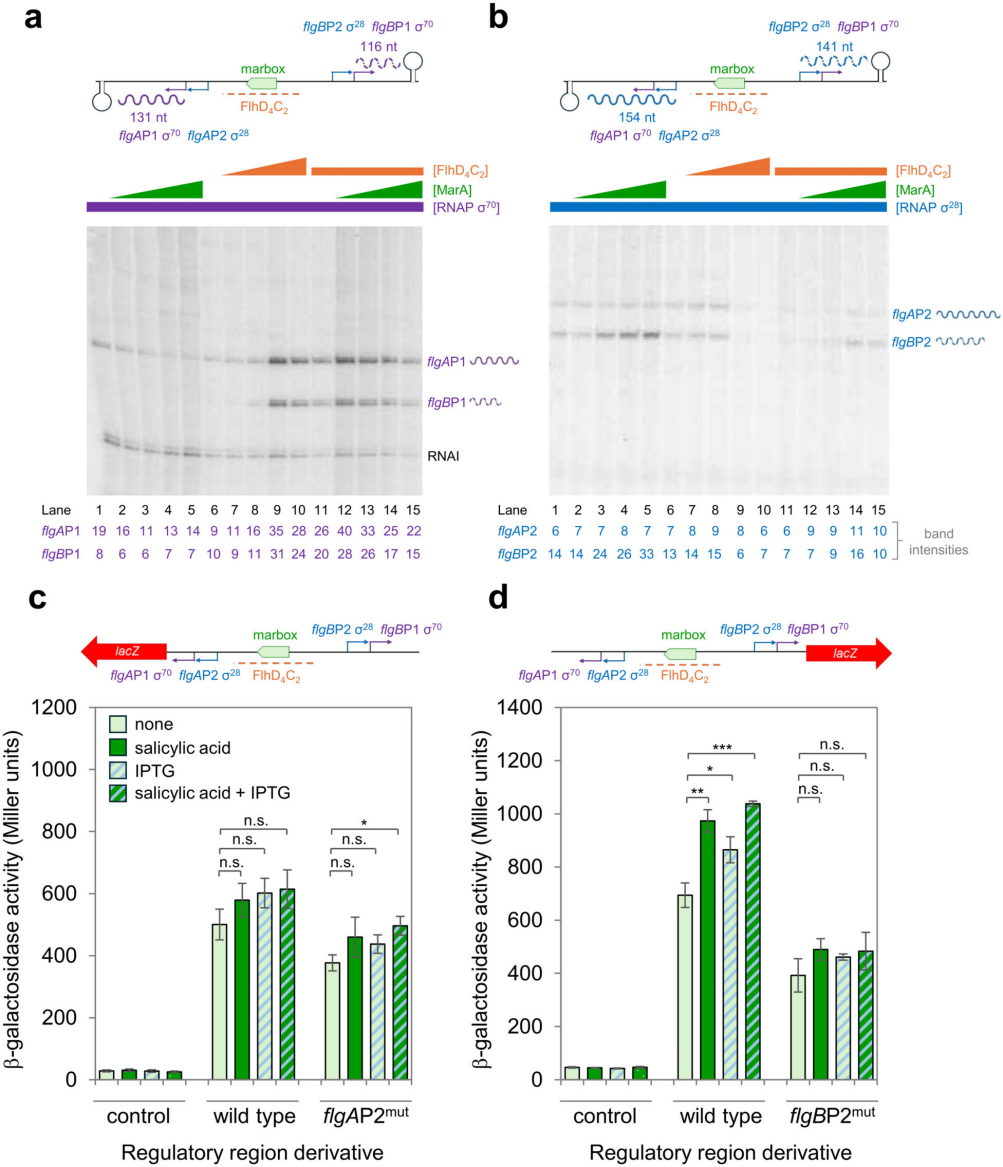
MarA activates transcription of *flgB* from a σ^28^-dependent promoter. (**a**) The σ^70^-dependent promoters *flgA*P1 and *flgB*P1 are activated by FlhD_4_C_2_, but not MarA, *in vitro*. Results of *in vitro* transcription assays to monitor σ^70^-dependent transcription from *flgA*P1 and *flgB*P1. The RNAI transcript is derived from the plasmid replication origin and serves as an internal control. RNA polymerase was used at a concentration of 0.15 μM. Where present, MarA was added at concentrations of 1, 2 , 4, and 5 µM. We used FlhD_4_C_2_ at concentrations of 0.05, 0.1, 0.2, and 0.25 µM. In lanes 11–15, FlhD_4_C_2_ was used at 0.2 µM concentration. Note that higher transcription factor concentrations can sometimes result in lower overall levels of transcription, indicated by a reduction in RNAI levels. This is likely due to non-specific DNA binding. (**b**) The σ^28^-dependent promoters *flgA*P2 and *flgB*P2 are repressed by FlhD_4_C_2_, and the latter activated by MarA, *in vitro*. As in panel (a) except that σ^28^-associated RNA polymerase was used. Note that this version of RNA polymerase cannot generate the RNAI transcript. (**c**) The σ^28^-dependent *flgA*P2 promoter is not regulated by MarA *in vivo*. Results of β-galactosidase assays using T7 express cells carrying pRW50 or derivatives with the indicated *flgA::lacZ* fusions. Cells also encoded pET21a-*fliA* to provide low levels of σ^28^ due to leaky expression. Cells were grown in LB medium supplemented with salicylic acid (5 µM), to induce MarA expression, or IPTG (1 µM) to induce a short burst of high level of σ^28^ production. The results shown are the mean of three independent experiments with error bars showing standard deviation. A two-tailed homoscedastic Student’s *t*-test was used to calculate *P* where appropriate; otherwise, a two-tailed test was used (* <.05, ** <.01, and *** <.001). (**d**) The σ^28^-dependent *flgB*P2 promoter is activated by MarA *in vivo*. As in panel (c) except that *flgB::lacZ* fusions were used. *P* was calculated as in panel (c).

Transcription from *flgA*P1 and *flgB*P1 is dependent on σ^70^ and activated by FlhD_4_C_2_ [45]. Since MarA and FlhD_4_C_2_ compete to bind the regulatory region (Fig. 2d), the stimulatory effect of MarA observed *in vivo* is counter intuitive (Fig. 1d). To better understand the roles of MarA, σ^70^-bound RNA polymerase, and FlhD_4_C_2_, we used *in vitro* transcription assays. To create a DNA template for transcription, the DNA region between *flgAMN* and *flgBCDEFGHIJ/KL* was cloned in plasmid pLSR. This places the divergent promoters between λ*oop* terminator sequences [47]. Transcription requiring σ^70^, from *flgA*P1 and *flgB*P1, is expected to generate transcripts 131 and 116 nucleotides in length, respectively. A schematic representation of the construct is shown in Fig. 3a (top panel). Assay results using σ^70^-associated RNA polymerase, and different combinations of regulatory proteins, are shown below the schematic. The RNAI transcript, generated from the plasmid replication origin, serves as an internal control. In the absence both FlhD_4_C_2_ and MarA, only transcription from *flgA*P1 is evident (lane 1). Addition of increasing MarA concentrations reduces this transcription (lanes 2–5). As expected, FlhD_4_C_2_ substantially increased transcription from both *flgA*P1 and *flgB*P1 (lanes 6–10). We conclude that the former promoter has low level intrinsic activity and the latter is completely dependent on FlhD_4_C_2_. In the presence of FlhD_4_C_2_, addition of MarA had no impact on transcription from either promoter relative to RNAI (lanes 11–15). Most likely, this is because MarA and FlhD_4_C_2_ compete for binding the same site. Taken together, the data suggest that activation by MarA *in vivo* is either indirect or not σ^70^ mediated via *flgA*P1 and *flgB*P1.

### The intergenic region between *flgAMN* and *flgBCDEFGHIJ/KL* contains divergent σ^28^-dependent promoters

To understand whether the regulatory region might contain other promoters, we manually searched the nucleotide sequence. We noticed regions closely resembling the −10 and −35 promoter elements recognized by the alternative σ^28^ subunit of RNA polymerase [35]. Underlined blue in Fig. 2a, and shown in Fig. 3a schematic, the predicted promoters share the same −35 hexamer. For comparison, the consensus σ^28^ binding motif is shown in Fig. 2b (bottom panel) [35]. We named the promoters *flgA*P2 and *flgB*P2. To assess activity, each promoter was individually cloned upstream of the λ*oop* terminator in plasmid pSR. The resulting constructs were used as templates for *in vitro* transcription with σ^28^-bound RNA polymerase. We also tested derivatives of each construct where the predicted −10 element for σ^28^ was mutated. The data are shown in Supplementary Fig. S1. Consistent with our predictions, the expected mRNAs were produced only if the −10 element was intact.

### The σ^28^-dependent *flgB*P2 promoter is activated by MarA *in vitro*

In the context of pLSR, the *flgA*P2 and *flgB*P2 promoters are expected to generate transcripts 154 and 141 nucleotides in length, respectively (Fig. 3b, top panel). To understand the impact of MarA, we repeated our *in vitro* transcription experiment using σ^28^-bound RNA polymerase. The results are shown below the schematic in Fig. 3b. Note that σ^28^-bound RNA polymerase cannot generate the RNAI transcript [28]. Consistent with our prediction, transcripts of the expected length were observed (lane 1). Addition of MarA did not alter transcription from *flgA*P2 but increased transcription from *flgB*P2 (lanes 2–5). Note that the binding site for FlhD_4_C_2_ completely overlaps both σ^28^-dependent promoters (Fig. 2a). Consistent with this, addition of FlhD_4_C_2_ repressed transcription (Fig. 3b, lanes 6–10). In the presence of FlhD_4_C_2_, MarA was able to stimulate transcription from *flgB*P2, but to a lesser extent than observed with the naked DNA template (compare lanes 1–5 with 11–15). We conclude that, in addition to direct activation of *flgB*P2, MarA indirectly activates by competing with FlhD_4_C_2_. Conversely, MarA has no direct stimulatory effect on transcription from *flgA*P2 *in vitro*. Hence, increased *flgA* transcription, dependent on MarA *in vivo*, must be indirect (Fig. 1d).

### MarA activates σ^28^-dependent transcription from *flgB*P2 *in vivo*

Overall, our *in vitro* transcription data are consistent with the presence of secondary σ^28^-dependent promoters for both *flgAMN* and *flgBCDEFGHIJ/KL*. The latter of these promoters is activated by MarA, and both are subject to direct repression by FlhD_4_C_2_. With these observations in mind, we reappraised our β-galactosidase assay data in two ways. First, expression of σ^28^ requires FlhD_4_C_2_. Hence, in the absence of FlhD_4_C_2_, it is unsurprising that the impact of MarA on *flgB* transcription from the regulatory region is small (Fig. 1c). Second, although σ^28^ is expressed upon production of FlhD_4_C_2_, the latter should repress both *flgA*P2 or *flgB*P2, while switching on σ^70^-dependent transcription from *flgA*P1 and *flgB*P1. Hence, to better understand *flgA*P2 and *flgB*P2 *in vivo*, we sought to uncouple σ^28^ expression from FlhD_4_C_2_. To do this, we exploited the known phenomenon of leaky gene expression from isopropyl β-D-1-thiogalactopyranoside (IPTG) inducible promoters reliant on the T7 phage RNA polymerase [48]. We transformed cells with both pET21a, encoding σ^28^, and pRW50, having the *flgA–flgB* intergenic region fused to *lacZ* in either possible orientation. To induce MarA production, we added salicylic acid to cultures. We also tested the impact of brief σ^28^ induction with IPTG. The results are shown in Fig. 3c and d. Consistent with our *in vitro* transcription assays, induction of MarA had little effect on *flgA* expression dependent on σ^28^ (Fig. 3c). Furthermore, any small differences were also observed when *flgA*P2 was mutated. Conversely, for expression of *flgB*, addition of salicylic acid significantly increased *lacZ* expression, and this effect was abolished when *flgB*P2 was deleted (Fig. 3d). We conclude that *flgB*P2 is a secondary promoter of *flgB* transcription, dependent on σ^28^, and activated by MarA. This is intriguing since all known MarA-activated promoters are σ^70^-dependent and have MarA binding sites at specific positions upstream of the core promoter [21, 22, 25, 27]. On the contrary, the σ^28^-dependent promoter *flgB*P2 is stimulated by MarA binding a site between the −10 and −35 elements. This suggests a different mechanism of activation.

### The *flgB*P2 −10 element is incompletely unwound by σ^28^ during transcription initiation

**Figure 4.**
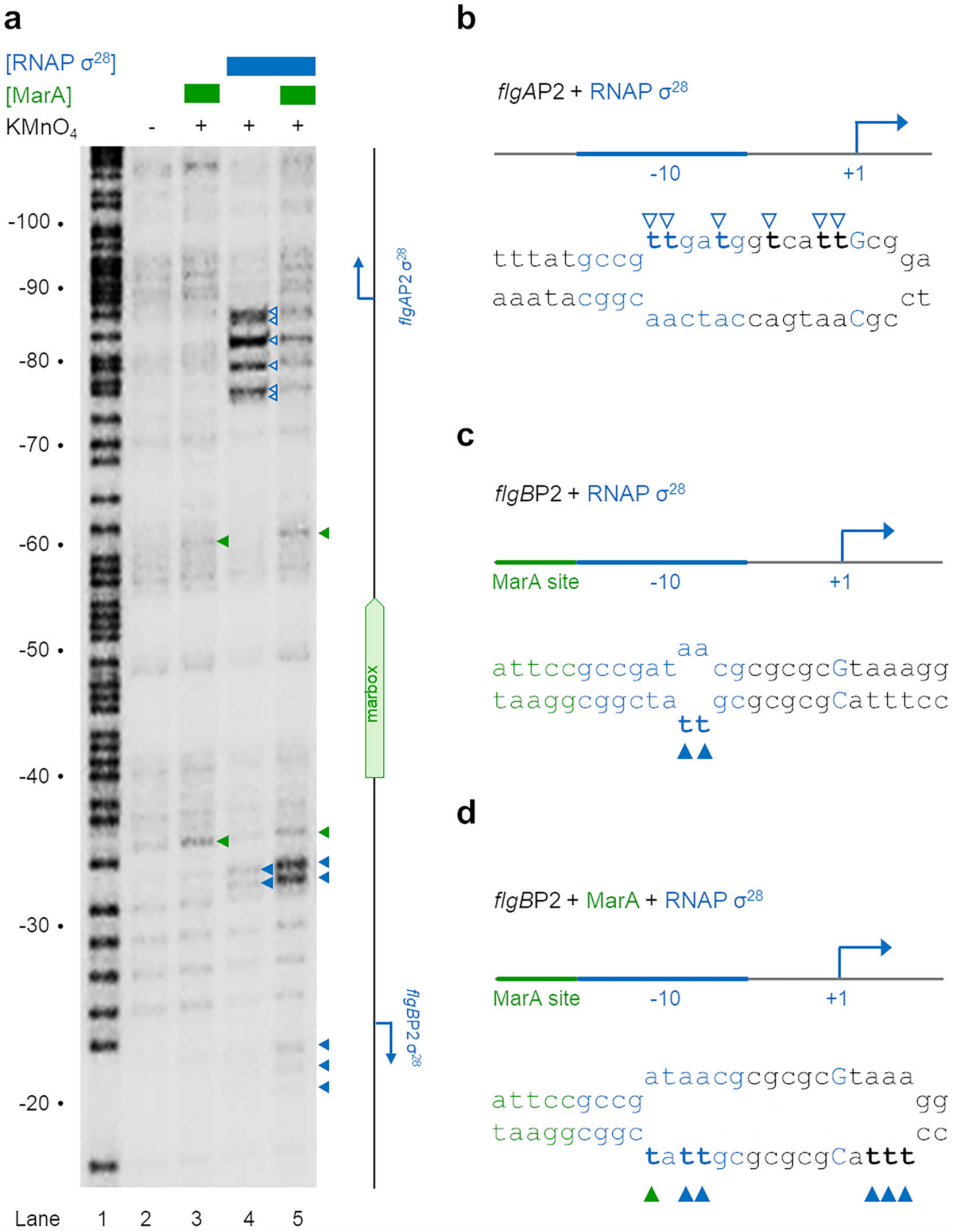
A defect in opening of the *flgB*P2 promoter is corrected by MarA-induced base pair distortions. (**a**) Results of a KMnO_4_ footprint with different combinations of MarA and σ^28^-associated RNA polymerase. Reactivity to KMnO_4_ due to DNA melting at *flgA*P2 and *flgB*P2 is shown by open and closed blue triangles, respectively. Equivalent reactivity changes due to MarA are indicated by green arrows. The gel is calibrated with a Maxam–Gilbert sequencing reaction numbered according to the position of the *flgB*P1 TSS. The TSS locations for *flgA*P2 and *flgB*P2 are shown in the adjacent schematic. (**b**) Schematic representation of DNA opening at *flgA*P2 induced by σ^28^-associated RNA polymerase alone. The schematic shows the DNA strand base sequences for *flgA*P2 and regions of DNA unwinding indicated in lane 4 of panel (a). The thymine bases reactive to KMnO_4_, in the presence of σ^28^-bound RNA polymerase, are marked by triangles. The promoter −10 element is in blue and the transcription start site (+1) is in upper case and marked by a bent arrow. (**c**) Schematic representation of DNA opening at *flgB*P2 induced by σ^28^-associated RNA polymerase alone. As in panel (b) except that the *flgB*P2 sequence and opening pattern (in the absence of MarA) are shown. Note that a portion of the MarA site (green) is also highlighted. (d) Schematic representation of DNA opening at *flgB*P2 induced by MarA and σ^28^-associated RNA polymerase. As in panel (b) except that data are shown for reactions with MarA. The position of KMnO_4_ reactivity induced by MarA is highlighted by a green triangle.

Activators in the MerR family bind between promoter −10 and −35 elements that are too far apart, kink the DNA, and so bring the core promoter sequences into correct juxtaposition [17, 18]. This seems unnecessary at *flgB*P2; the two hexamers for σ^28^ binding are optimally separated (Fig. 2). Hence, MarA must correct a different transcription initiation defect. We turned our attention to the subsequent duplex unwinding step, which can be detected by KMnO_4_ footprinting. Specifically, we measured the ability of σ^28^-bound RNA polymerase to form open complexes with *flgA*P2 and *flgB*P2. Figure 4a shows the resulting DNA cleavage patterns. Note that, for consistency, the gel calibration is numbered with respect to the *flgB*P1 TSS. The location of each σ^28^-dependent TSS is shown in the schematic adjacent to the gel image. As expected, addition of σ^28^-bound RNA polymerase disrupted base pairing around the −10 elements for *flgA*P2 and *flgB*P2 (lane 4, indicated by open and closed triangles, respectively). However, the patterns of DNA opening were strikingly different. At *flgA*P2, the DNA duplex is fully opened (see schematic in Fig. 4b). Conversely, we detected only partial, and very inefficient, opening of *flgB*P2; only positions −9 and −8, in the middle of the −10 element, were subject to KMnO_4_ attack (Fig. 4c). Of note, we did not detect unwinding at position −11. Since promoter unwinding is nucleated by RNA polymerase capture of A_−11_, we conclude that this step is compromised in the context of *flgB*P2.

### Distortion of promoter base pairing by MarA permits complete *flgB* promoter opening by σ^28^

To understand whether MarA could correct the *flgB*P2 unwinding defect, we repeated our assay in the presence of MarA. As described above, with MarA alone, we observed DNA base pair distortions 5 bp on either side of the MarA site (Fig. 4a, compare lanes 2 and 3, marked by green triangles). Strikingly, one of these locations is *flgB*P2 base pair −11 (see green triangles in Fig. 2a). We reasoned that this MarA-induced DNA conformation change might stimulate transcription from *flgB*P2. Specifically, capture of non-template base A_−11_ by σ^28^ might be improved. In turn, this should facilitate complete DNA unwinding [4, 49]. Consistent with this, in the presence of both MarA and σ^28^ we observed complete unwinding of the *flgB*P2 −10 element, from the upstream edge at position −11 to position +5 downstream of the TSS (Fig. 4a, lane 5 and Fig. 4d). We conclude that MarA may activate *flgB*P2 by distorting the base pair at position −11, allowing σ^28^ RNA polymerase to fully open the duplex DNA. Interestingly, activation of *flgB*P2 coincides with reduced opening of *flgA*P2 (compare lanes 4 and 5). Since the two promoters share the same −35 element (Fig. 2a), we speculate that this is due to competition between RNA polymerase molecules.

### Artificial distortion of base pairing at the *flgB* promoter removes the requirement for MarA

**Figure 5.**
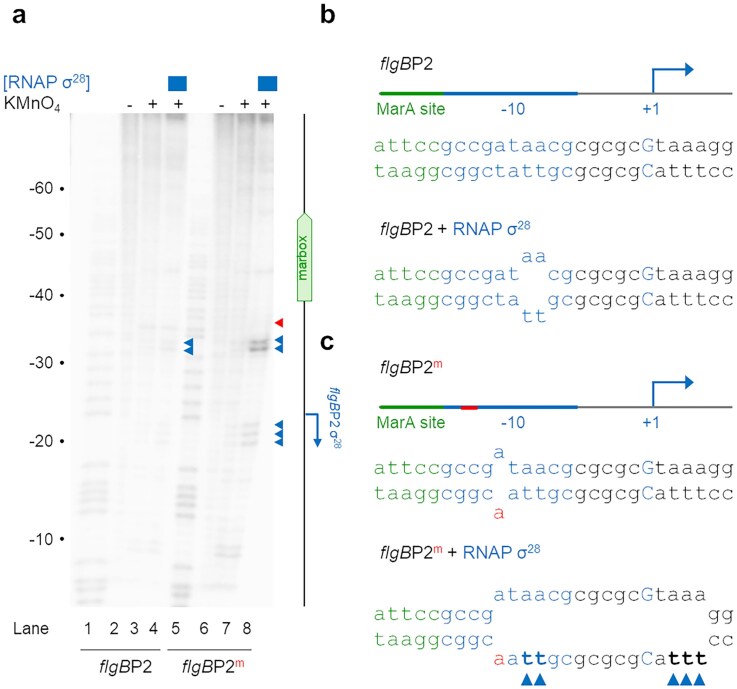
Artificial disruption of *flgB*P2 promoter base pairing removes the requirement for MarA. (**a**) Results of a KMnO_4_ footprint with σ^28^-associated RNA polymerase and variants of *flgB*P2. Reactivity to KMnO_4_, due to DNA melting at *flgB*P2, is shown by closed blue triangles. The *flgB*P2^m^ variant (lanes 6–8) has a single base mismatch that prevents base pairing at the location indicated by the red triangle. This is the same base pair that MarA protein perturbs upon binding the DNA. The gel is calibrated with a sequencing reaction and numbering is with respect to the *flgB*P1 TSS. (**b**) Schematic representation of the wild type *flgB*P2 sequence. The top and bottom panels indicate the degree of DNA unwinding in the presence and absence of σ^28^-bound RNA polymerase [equivalent to panel (a) lanes 3 and 4]. Triangles indicate that the thymine bases reactive to KMnO_4_ in the presence of RNA polymerase bound with σ^28^. **(c)** Schematic representation of an *flgB*P2^m^ variant having single base pair mismatch to facilitate pre-opening of the DNA. The top and bottom panels indicate the degree of DNA unwinding in the presence and absence of σ^28^-bound RNA polymerase [equivalent to panel (a) lanes 7 and 8]. The altered nucleotide is shown in red. Triangles indicate that the thymine bases reactive to KMnO_4_ in the presence of RNA polymerase bound with σ^28^.

Our model predicts that the requirement for MarA, at *flgB*P2, should be removed if A_−11_ cannot base pair because of a single nucleotide mismatch on the template DNA strand. To test this, we generated oligonucleotides corresponding to template and non-template strand *flgB*P2 sequences. Critically, while the non-template strand retained the wild type A_−11_ base, the opposing template strand thymine was replaced with adenine. Hence, when the oligonucleotides are annealed, bases at position −11 should remain unpaired in the *flgB*P2^m^ variant. To understand the consequences, we repeated our KMnO_4_ footprinting analysis. As expected, with the wild type promoter, σ^28^-associated RNA polymerase was unable to efficiently open the DNA (Fig. 5a, lanes 2–4). Conversely, in the presence of the −11 mismatch, a complete open complex was generated (lanes 6–8). Note that, although the bp at position −11 must be unpaired, this is not detected in the footprinting assay; the non-template strand thymine is replaced by adenine, which is much less reactive to KMnO_4_. As expected, *flgB*P2^m^ variant was not subject to activation by MarA (Supplementary Fig. S2).

### Structural modelling of the complex formed by *flgB*P2, σ^28^-bound RNA polymerase, and MarA

**Figure 6.**
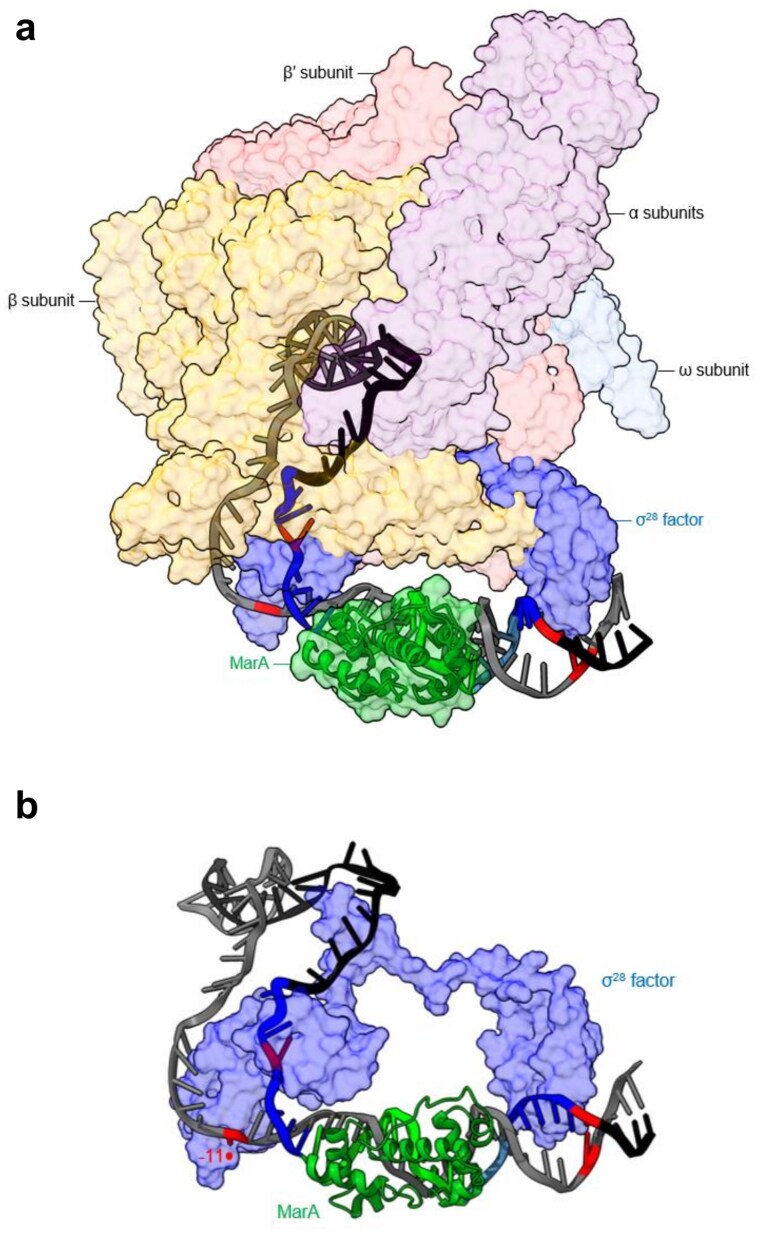
Structural modelling of the ternary complex formed between *flgB*P2, MarA, and σ^28^-associated RNA polymerase. (**a**) Structural model of MarA and the σ^28^ RNA polymerase holoenzyme in the context of the *flgBP2* open complex. The σ^28^ factor is coloured blue, and labelled alongside other RNA polymerase subunits, while MarA is shown as green. Where needed, proteins are partially transparent to allow DNA (grey) trajectory to be visualized. The promoter −10 and −35 elements recognized by σ^28^ are in blue on the top strand and base pairs distorted upon MarA binding are highlighted red. (**b**) An expansion with only MarA and σ^28^ visible. Promoter elements are labelled. Note that the −10 element base pair, at position −11, forms the upstream boundary of DNA opening and is targeted for distortion by MarA.

To understand how MarA and σ^28^-bound RNA polymerase might co-bind *flgB*P2, we used models generated from structures of DNA-bound MarA [34] and σ^28^ holoenzyme [49]. The *flgB*P2 promoter sequence was aligned with equivalent DNA features of the latter structure. This allowed us to determine the location at which MarA should be positioned. Having positioned MarA, we introduced a 35° DNA bend known to be induced upon MarA binding [34]. The final model is shown in Fig. 6. The RNA polymerase and MarA molecules are offset by ~90°, around the helical axis of the DNA molecule, with respect to each other. The base pairs deformed by MarA, in KMnO_4_ footprinting assays, are highlighted red. The non-template strand −10 and −35 element sequences are shown in green. As expected, the upstream boundary of the open complex is one of the two base pairs subject to perturbation by MarA. The model suggests that MarA makes no direct contacts with core RNA polymerase, or σ^28^, in this context.

## Conclusions

In bacteria, most transcriptional activators stabilize the interaction between RNA polymerase and the promoter DNA [1]. Depending on the order in which these ternary complexes assemble, the process may be referred to as recruitment or pre-recruitment [12–15]. Usually, this is achieved by simultaneous interactions between the molecular entities [1]. Less frequently, an activator may kink the DNA to correctly align promoter elements recognized by RNA polymerase [17, 18]. Activation can also be indirect and result from displacement of a repressor protein or inhibition of transcriptional interference [50, 51]. In this work, we have discovered a mechanism of transcription activation not previously reported. We show that the MarA protein, primarily an activator by pre-recruitment at σ^70^-dependent promoters [52], can also stimulate transcription requiring σ^28^. In the latter case, the mode of activation is different; MarA binds within, rather than upstream of, the core promoter. Furthermore, we find no evidence of a critical interaction with the RNA polymerase σ^28^ factor. Instead, our data are consistent with distortions to the DNA duplex, induced by MarA, correcting a defect in *flgB*P2 −10 element opening by σ^28^. Hence, by altering DNA base pairing, MarA facilitates duplex opening and transcription levels increase. To our knowledge, no other DNA binding regulators have been shown to activate transcription in this way. We do not rule out the possibility that changes in DNA conformation, induced by MarA, might also enhance promoter affinity for RNA polymerase.

Interestingly, as many proteins can distort the nucleic acid duplex, we suggest that other promoters will be subject to similar control. For example, although not linked to a promoter opening defect, integration host factor (IHF) destabilizes −10 element base pairing at the *ilv*P_G_ promoter [53]. While recognized by σ^70^, that unwinds DNA adeptly, the *ilv*P_G_ −10 hexamer lacks a consensus T_−7_ base on the non-template DNA strand. Like A_−11_, base T_−7_ flips out of the base stack and sits within a σ^70^ pocket, as duplex unwinding proceeds towards the TSS [4, 5]. Thus, we predict IHF acts to overcome this missing feature of *ilv*P_G_.

We were initially surprised that, at the intergenic region between *flgAMN* and *flgBCDEFGHIJ/KL*, the σ^28^ holoenzyme can use *flgA*P2, but not *flgB*P2, unaided. On reflection, this is probably because the *flgB*P2 −35 element is particularly poor (Fig. 2) and σ^28^ requires promoters closely matching the consensus [11]. Importantly, this stringency reflects the complete absence of a W-dyad, and associated DNA melting defects, in σ^28^ [11]. Thus, mutations that create a partial W-dyad in σ^28^ allow sub-optimal promoters to be used [11]. We argue that, by destabilizing the base pair at promoter position −11, MarA mimics the impact of such σ^28^ mutations. Thus, *flgB*P2 becomes a viable σ^28^ target if MarA is bound. Since most alternative σ factors have DNA melting defects, activation by DNA distortion could be more common at associated promoters. Indeed, there may be many examples of cryptic promoters only recognized by alternative σ factors if nucleic acid structure is first perturbed.

The full role of MarA in controlling the flagellar cascade is unclear. It seems unlikely that MarA upregulates *flgAMN* and *flgBCDEFGHIJ/KL* unless (i) the regulatory cascade, including σ^28^ expression, has already been activated by FlhD_4_C_2_ and (ii) FlhD_4_C_2_ levels have subsequently fallen [35, 36]. The latter would be necessary to allow MarA binding at the *flgB*P2 promoter region that can also be bound by FlhD_4_C_2_. This may explain why a previous analysis of global σ^28^ binding did not identify *flgA*P2 and *flgB*P2 [35]. We speculate that, when already motile cells encounter stress, MarA may boost the expression of some flagellar system components. This might permit a cell to repair or replace damaged and ejected flagella upon antibiotic exposure. Overall, this model is consistent with our previous observations. In particular, we have shown that MarA activates expression of the *ycgZ–ymgABC* operon and represses *csgD*, to prevent biofilm formation [23, 27]. Thus, production of MarA could favour a motile lifestyle in some scenarios. Previously, we suggested that MarA might not inhibit genes for biofilm formation if the pathway had already been initiated. With this in mind, it is interesting to consider the previous work of Thota and Chubiz, who suggested MarA might directly repress transcription of *flhDC* in *Salmonella enterica* serovar Typhimurium [54]. Hence, it is tempting to infer that MarA acts to repress the flagellar cascade unless it has already been initiated. In the latter case, boosting motility may offer the best chances of survival.

## Funding

K.M.K. is grateful to the Darwin Trust of Edinburgh for award of a PhD studentship. D.C.G. would like to acknowledge the Wellcome Trust for investigator award 212193/Z/18/Z. Open access charges were paid by the University of Birmingham.

## Supplementary Material

gkaf1424_Supplemental_File

## Data Availability

Full gel images are available in Supplementary Fig. S3. Raw data for graphs are available on request.

## Notes

Present address: Faculty of Medical Sciences, Newcastle University, Newcastle upon Tyne, NE2 4HH, United Kingdom
