# Supplementary material for: Activation of bacterial transcription by distortion of promoter base pairing
Source: Nucleic Acids Res. 2026 Jan 8;54(1):gkaf1424. doi: 10.1093/nar/gkaf1424 (PMC12781870; doi:10.1093/nar/gkaf1424)
Supplement: gkaf1424_Supplemental_File [file gkaf1424_supplemental_file.pdf]

Figure S1

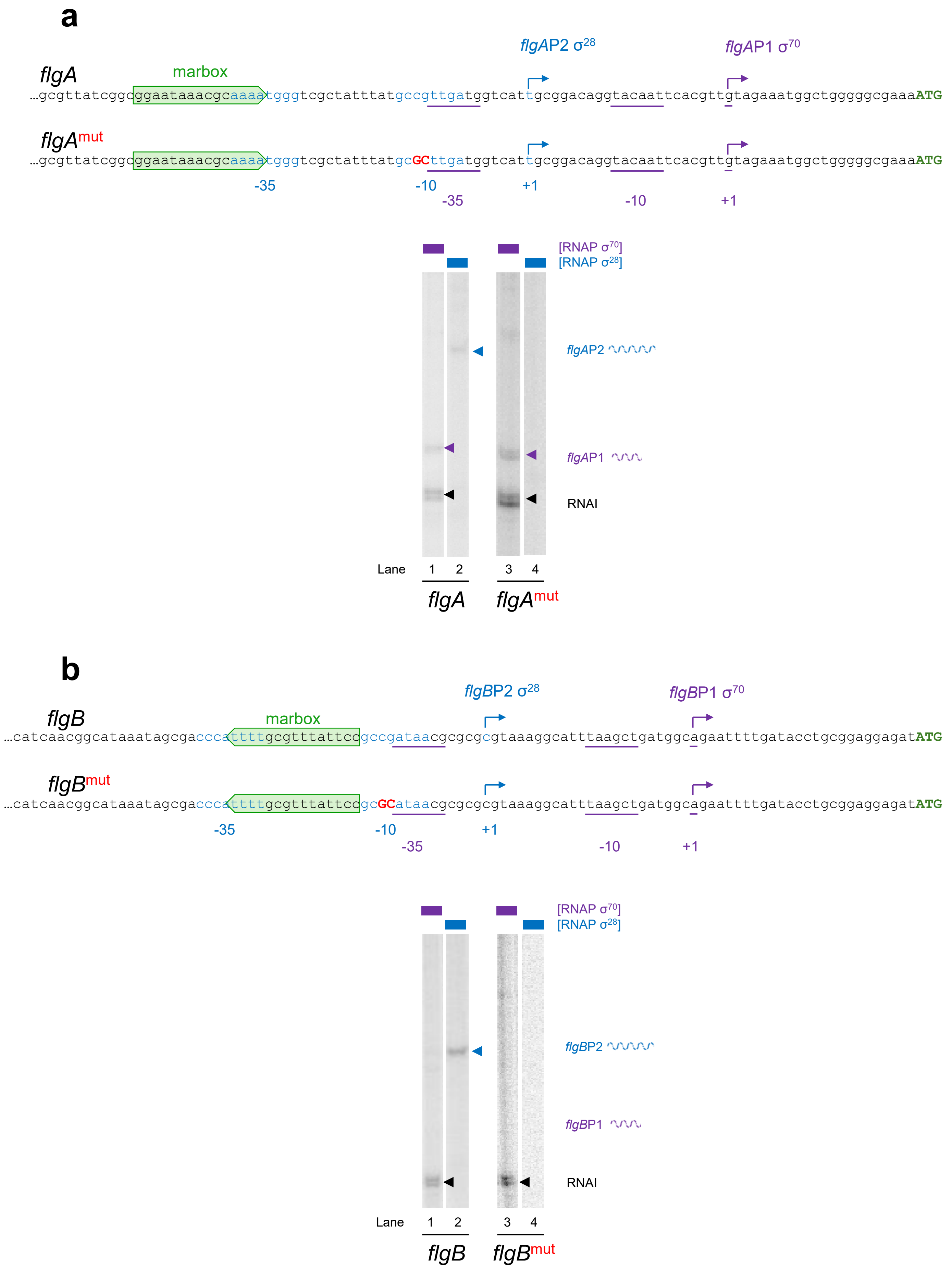
a
flgAP2 σ28
flgAP1 σ70
flgA
marbox
…gcgttatcggcggaataaacgcaaaatgggtcgctatttatgccgttgatggtcattgcggacaggtacaattcacgttgtagaaatggctgggggcgaaaATG
flgAmut
…gcgttatcggcggaataaacgcaaaatgggtcgctatttatgcGCttgatggtcattgcggacaggtacaattcacgttgtagaaatggctgggggcgaaaATG
-35
-10
+1
-35
-10
+1
[RNAP σ70]
[RNAP σ28]
flgAP2
flgAP1
RNAI
Lane
1
2
3
4
flgA
flgAmut
b
flgBP2 σ28
flgBP1 σ70
flgB
marbox
…catcaacggcataaatagcgacccattttgcgtttattccgccgataacgcgcgcgtaaaggcatttaagctgatggcagaattttgatacctgcggaggagatATG
flgBmut
…catcaacggcataaatagcgacccattttgcgtttattccgcGCataacgcgcgcgtaaaggcatttaagctgatggcagaattttgatacctgcggaggagatATG
-35
-10
+1
-35
-10
+1
[RNAP σ70]
[RNAP σ28]
flgBP2
flgBP1
RNAI
Lane
1
2
3
4
flgB
flgBmut

Figure S2

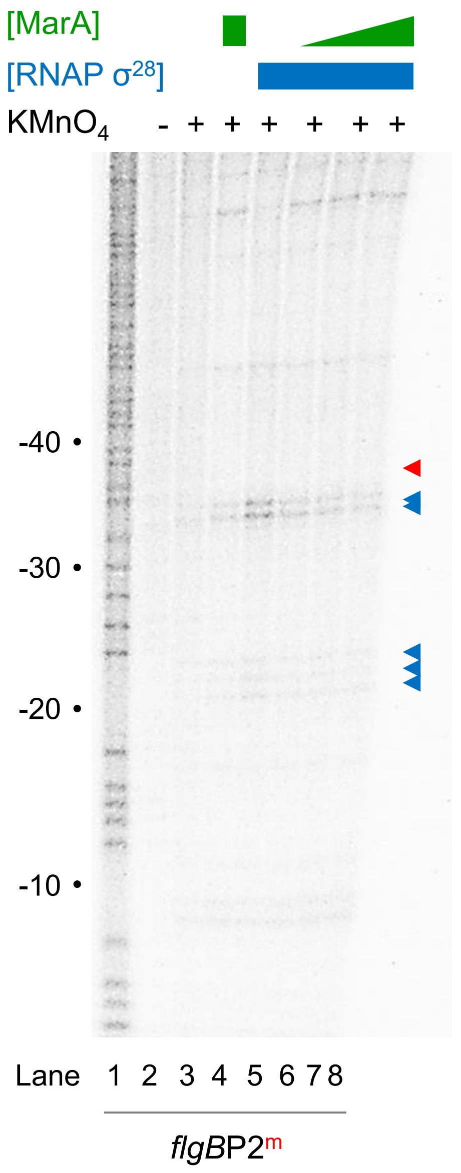

Figure S3

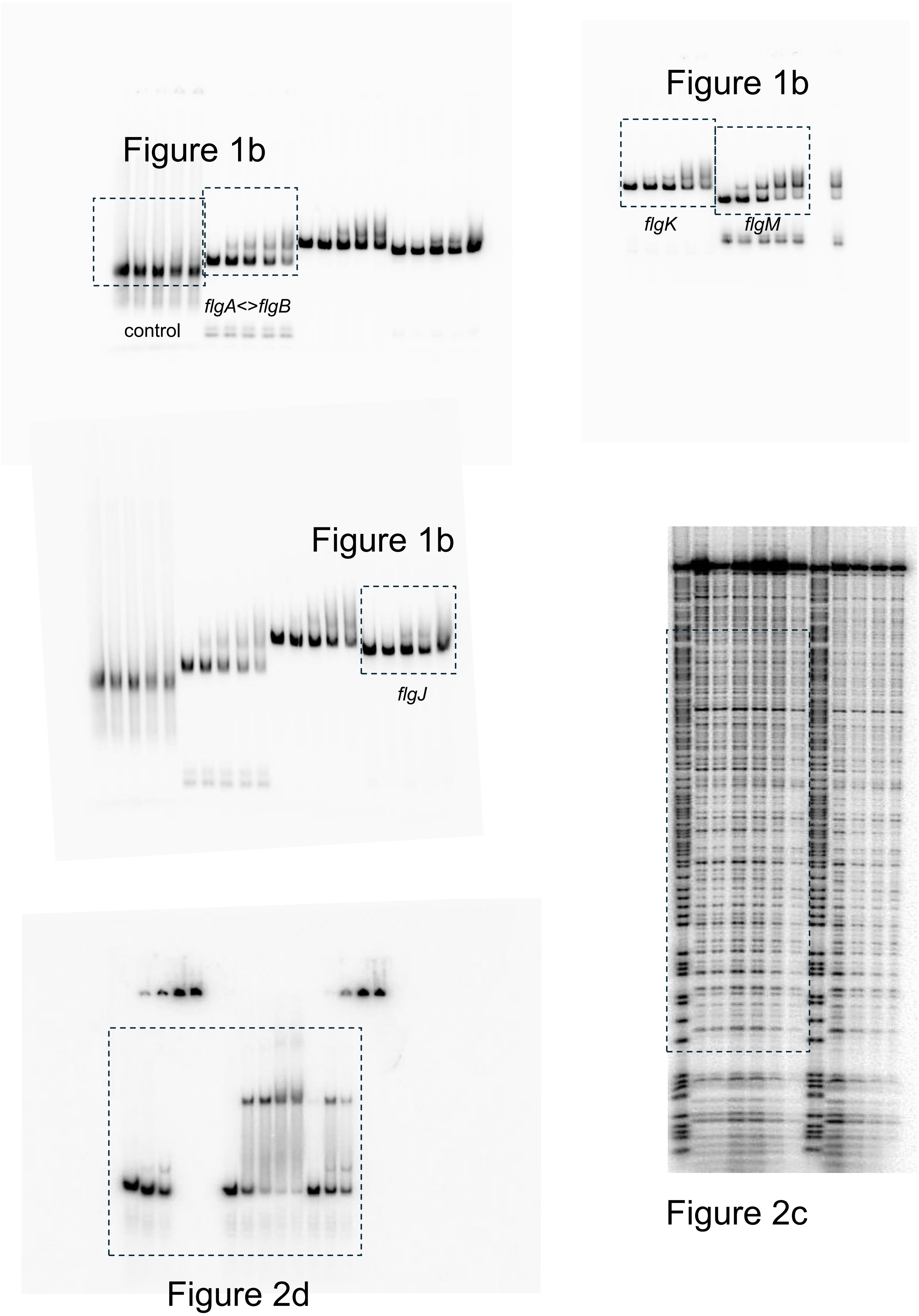

Figure 1b
control
flgA<>flgB
Figure 1b
flgK
flgM
Figure 1b
flgJ
Figure 2d
Figure 2c

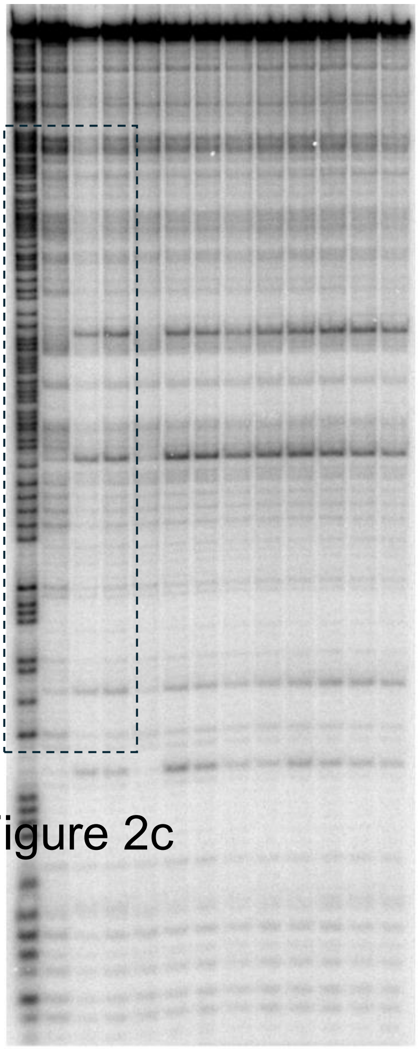
Figure 2c

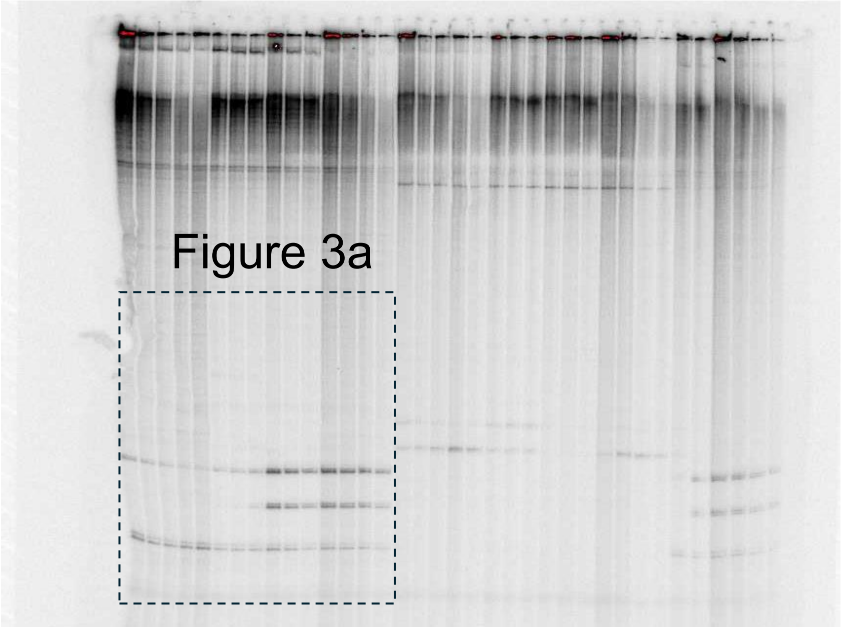
Figure 3a

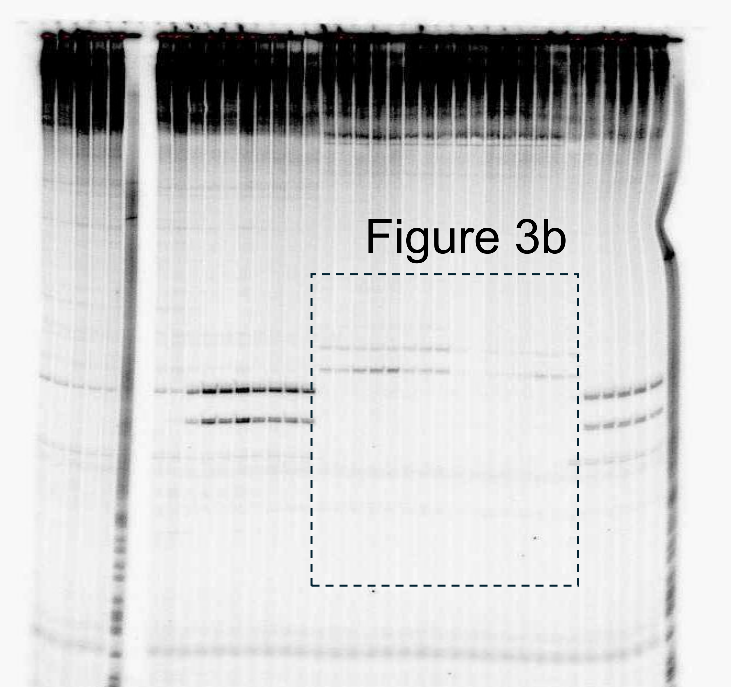
Figure 3b

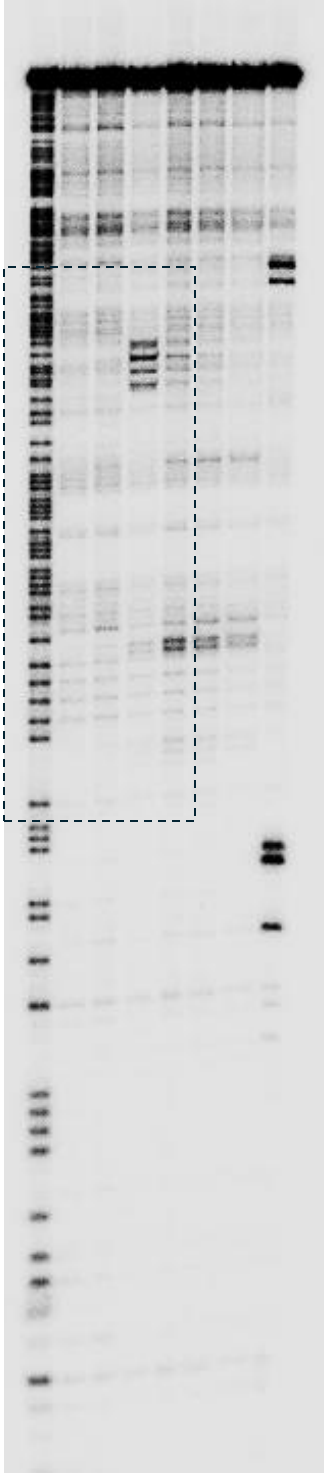
Figure 4a

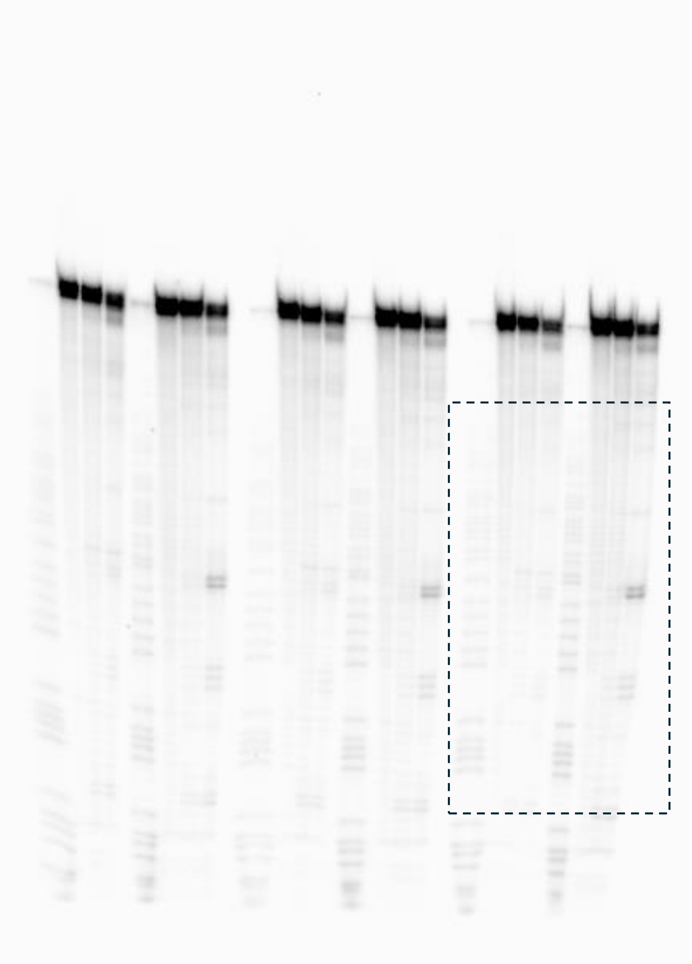
Figure 5a

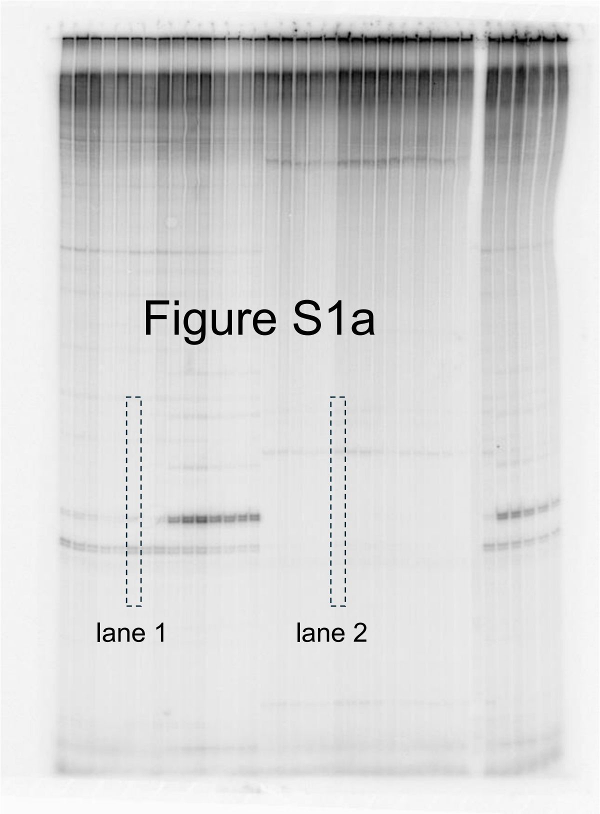
Figure S1a
lane 1
lane 2

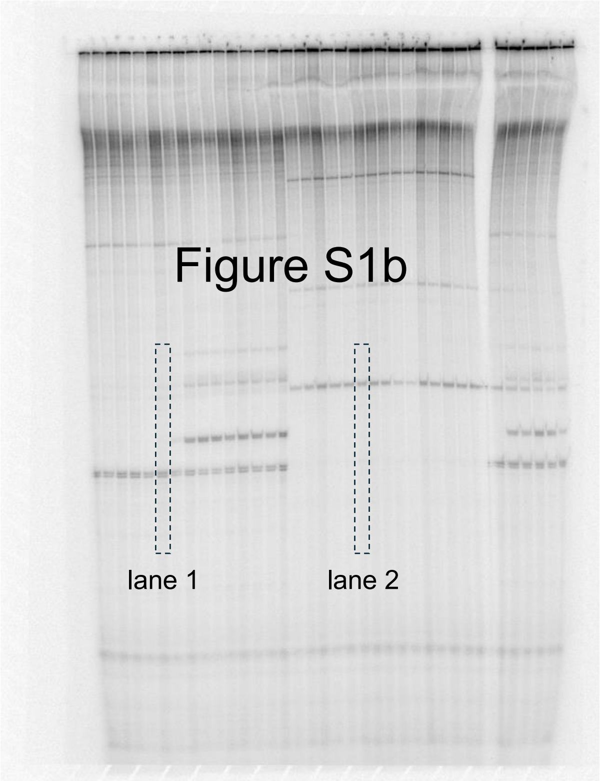
Figure S1b
lane 1
lane 2

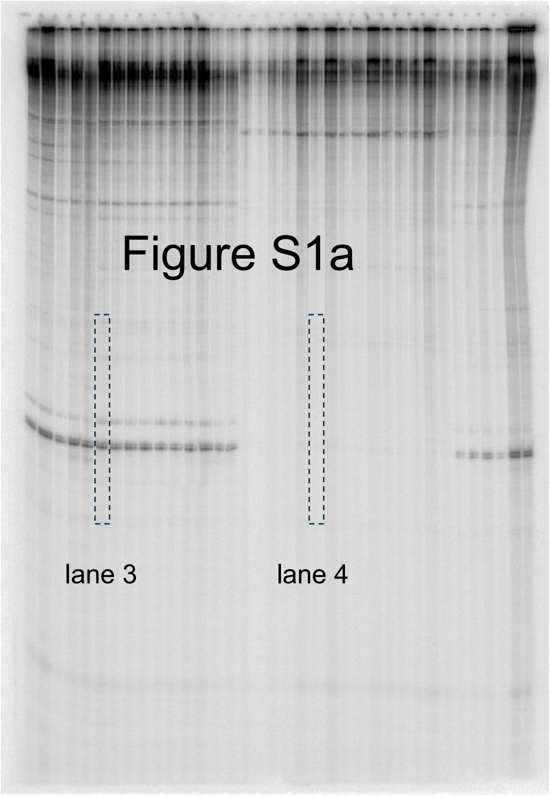
Figure S1a
lane 3
lane 4

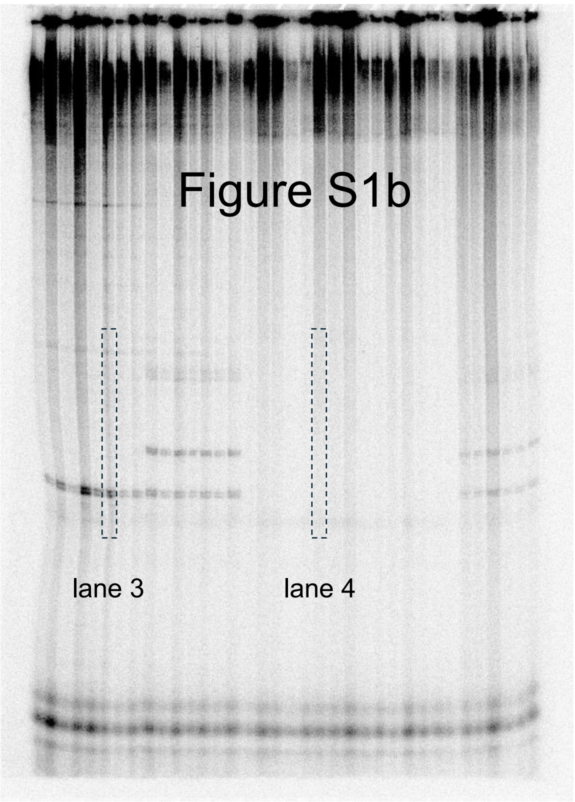
Figure S1b
lane 3
lane 4

SUPPLEMENTARY FIGURE LEGENDS

Figure S1: Identification of divergent σ^{28} dependent promoters in the *flgAB* intergenic region

a. Identification of *flgA*P2. The DNA sequence upstream of *flgA* (top) is shown with the proposed σ^{28} dependent *flgA*P2 promoter elements labelled. The transcription start site is shown as a bent arrow and labelled +1. The marbox is labelled and highlighted green. The *flgA*^{mut} sequence has the indicated point mutations (red and uppercase) in the *flgA*P2 -10 element. The lower gel lane images show results of *in vitro* transcription assays with each DNA fragment, and σ^{70} or σ^{28} RNA polymerase holoenzyme, cloned in plasmid pSR, upstream of a λ *oop* terminator. Transcripts derived from each promoter are labelled. The RNAI transcript serves as an internal control and is not transcribed by the σ^{28} holoenzyme.

b. Identification of *flgB*P2. The DNA sequence upstream of *flgB* (top) is shown with the proposed *flgB*P2 promoter elements labelled. The transcription start site is shown as a bent arrow and labelled +1. The marbox is labelled and highlighted green. The *flgB*^{mut} sequence has the indicated point mutations (red and uppercase) in the *flgB*P2 -10 element. The lower gel lane images show results of *in vitro* transcription assays with the wild type or mutated *flgB* regulatory fragments, and σ^{70} or σ^{28} RNA polymerase holoenzyme, cloned in plasmid pSR upstream of a λ *oop* terminator. Transcripts derived from each promoter are labelled.

Figure S2: MarA does not activate transcription from *flgB*P2^{m}

Reactivity to $KMnO_4$ due to DNA melting at *flgB*P2 is shown by blue triangles. The gel is calibrated with a Maxam-Gilbert sequencing reaction numbered according to the position of the *flgB*P1 transcription start site.

Figure S3: Uncropped gel images

The approximate regions shown in each figure are boxed and labelled.

Table S1: Strains, plasmids and oligonucleotides

Name	Description	Source
	Escherichia coli strains	
JCB387	Δ*nirB* Δ*lac*	[1]
T7 Express	*fhuA*2 *lacZ*::T7 gene1 [*lon*] *ompT gal sulA*11 R(*mcr*73::miniTn10--T *etS*)2 [*dcm*] R(*zgb*-210::Tn10--TetS) endA1 Δ(*mcrCmrr*) 114::IS10	NEB
	Plasmids	
pRW50	Broad-host-range lac fusion vector for cloning promoters on *Eco*RI–*Hin*dIII fragments: contains the RK2 origin of replication and encodes TcR	[2]
pSR	pBR322-derived plasmid containing an *Eco*RI–*Hin*dIII fragment upstream of the λ*oop* transcription terminator	[3]
pET28a	pET28a Protein expression vector with T7lac promoter	Novagen
pJ203	Encodes ChlorR; pUC origin. Protein overexpression vector; used for MarA overexpression for β-galactosidase assays.	ATUM.
pJ203*marA*	pJ203 derivative encoding *marA* under the control of a constitutive promoter.	ATUM.
pLSR	Encodes AmpR, derived from pSR; ColE1 origin. Carrying two λoop transcription terminators.	[4]
pET21a	pET21a Protein expression vector with T7lac promoter. Carry an N-terminal T7-Tag sequence plus an optional C-terminal His-Tag sequence. Encodes AmpR.	Novagen
pUC19*flhDC*	An M13mp7-derivitive carring the *flhDC* operon between KpnI/HinDIII restriction sites. The *flhDC* operon is under the control of a constitutive promoter. *lacZa* and associated promoter elements removed. Encodes AmpR.	GeneScript
pET26b	pET26b Protein expression vector with T7lac promoter. Carries an N-terminal pelB signal sequence for potential periplasmic localization, plus optional C-terminal HisTag sequence. Encodes KanR.	Novagen

Oligonucleotides (sequence 5' to 3')

Oligonucleotides used for sequencing of candidate plasmid constructs

pET-F	GATTATGCGGCCGTGTAC	This work
pET-R	ATGCGTCCGGCGTAG	This work
pSR-F	CCATATATCAGGGTTATTGTCTC	This work
pSR-R	CATCACCGAAACGCGCGAGG	This work
pRW50-F	GTTCTCGCAAGGACGAGAATTTC	This work
pRW50-R	AATCTTCACGCTTGAGATAC	This work

Oligonucleotides used to generate a control for EMSA

*estA*_F	GGCTGC**GAATTC**TAACATGATGCAACTCACAAAAAAA ATAAAAAAATTGCAAAATCCGTTTAACTAATCT	[5]
*estA*_R	GCCCG**AAGCTT**CATGTTACCTCCCGTCATGTTGTTTC ACGGATATTTGAGATTAGTTAAACGGATTTTG	[5]

Oligonucleotides used for amplification of the flagellar promoter regions

*flgA*_F	GGCTG**GAATTC**CTGCCATCAGCTTAAATGCC	This work
*flgA*_R	GCCCG**AAGCTT**CATTTCGCCCCCCAGCCATTT	This work
*flgB*_F	GGCTG**GAATTC**CGCCCCCAGCCATTT	This work
*flgB*_R	GCCCG**AAGCTT**CATATCTCCTCCGCAG	This work
*flgJ*_F	GGCTG**GAATTC**CAGACTGTGGTTACTCCACAAAC	This work
*flgJ*_R	GCCCG**AAGCTT**CATCAGATGATTTCCAG	This work
*flgK*_F	GGCTG**GAATTC**CGCAAATCTCACCAACATG	This work
*flgK*_R	GCCCG**AAGCTT**CATGGAGGTTCCTTATAAGCC	This work
*flgM*_F	GGCTG**GAATTC**GGCGTTGTTGATGCAGATG	This work
*flgM*_R	GCCCG**AAGCTT**CATGGTTTATTCTCATTGAGGGC	This work

Megaprimers with mutated -10 regions in the flagellar promoters

flgA::(-10)σ^{28}mut_F	GGTCGCTATTTATGC**GC**TTGATGGTGATTGCG	This work
flgB::(-10)σ^{28}mut_F	TTGCGTTTATTCCGC**GC**ATAACGCGCGCGTAA	This work

Oligonucleotides annealed to created double stranded flgBP2 promoter fragments

flgB F biotin:	ATCAACGGCATAAATAGCGACCCATTTTGCGTT<br>TATTCCGCCGATAACGCGCGCGTAAAGGCATTT<br>CAGAACGCTCGGTTGCCGCCGGGCGTTTTTTATT	This work
flgB R mismatch:	AATAAAAAACGCCCGGCGGCAACCGAGCGTTCT<br>GAAATGCCTTTACGCGCGCGTTAACGGCGGAAT<br>AAACGCAAAATGGGTCGCTATTTATGCCGTTGAT	This work
flgB R wild type:	AATAAAAAACGCCCGGCGGCAACCGAGCGTTCT<br>GAAATGCCTTTACGCGCGCGTTATCGGCGGAAT<br>AAACGCAAAATGGGTCGCTATTTATGCCGTTGAT	This work

References:

1. Page L, Griffiths L, Cole JA. 1990. Different physiological roles of two independent pathways for nitrite reduction to ammonia by enteric bacteria. *Arch Microbiol* 154:349–354.
2. Lodge J, Fear J, Busby S, Gunasekaran P, Kamini NR. 1992. Broad host range plasmids carrying the *Escherichia coli* lactose and galactose operons. *FEMS Microbiol Lett* 95:271–276.
3. Kolb A, Kotlarz D, Kusano S, Ishihama A. 1995. Selectivity of the Escherichia coli RNA polymerase Eσ38 for overlapping promoters and ability to support CRP activation. *Nucleic Acids Res* 23:819–826.
4. Mohamed Samir El-Robh and Stephen J.W. Busby. The Escherichia coli cAMP receptor protein bound at a single target can activate transcription initiation at divergent promoters: a systematic study that exploits new promoter probe plasmids. Biochemical Journal, 368:835–843, DEC 2002. ISSN 0264-6021, 1470-8728. doi: 10.1042/bj20021003. URL https://dx.doi.org/10.1042/bj20021003.
5. Haycocks JR, Sharma P, Stringer AM, Wade JT, Grainger DC. The molecular basis for control of ETEC enterotoxin expression in response to environment and host. PLoS Pathog. 2015 Jan 8;11(1):e1004605. doi: 10.1371/journal.ppat.1004605. PMID: 25569153; PMCID: PMC4287617.
